# Type I Thanatophoric Dysplasia: Clinical Outcome in the Absence of Molecular Genetic Confirmation: A Case Report

**DOI:** 10.7759/cureus.103819

**Published:** 2026-02-18

**Authors:** Khadija Mesbah, Kaoutar Ettoini, Kawtar Khabbach, Yousra El Boussaadni, Abdallah Oulmaati

**Affiliations:** 1 Pediatrics, Centre Hospitalier Universitaire Mohammed VI de Tanger, Tangier, MAR

**Keywords:** fgfr3 gene, molecular confirmation, prenatal diagnose, skeletal dysplasia, thanatophoric dysplasia

## Abstract

Thanatophoric dysplasia (TD) is the most common lethal congenital skeletal dysplasia, characterized by severe micromelia, a narrow thorax, and frontal bossing due to activating mutations in the fibroblast growth factor receptor 3 (FGFR3) gene on chromosome 4p16.3. First described by Maroteaux and Lamy in 1967, it carries a near-uniform perinatal mortality from respiratory insufficiency. We present the case of a male newborn in whom the diagnosis of thanatophoric dysplasia was established during the antenatal period.

## Introduction

Thanatophoric dysplasia (TD) is a rare, lethal congenital skeletal dysplasia that belongs to the broader group of genetic disorders affecting bone growth and development. It is characterized by marked rhizomelic limb shortening, a narrow thoracic cage resulting in pulmonary hypoplasia, macrocephaly with frontal bossing, bowed femora, and platyspondyly [1]. The estimated incidence ranges from approximately 1 in 20,000 to 1 in 50,000 births. The term “thanatophoric,” derived from the Greek meaning “death bearing,” reflects the uniformly fatal course of the condition in the perinatal period, primarily due to severe respiratory insufficiency. Clinically, TD is divided into two subtypes. Type I, the more common form, presents with curved femora and relatively preserved cranial morphology, whereas Type II is distinguished by straight long bones and a characteristic cloverleaf skull (kleeblattschädel) caused by premature cranial suture fusion. TD results from activating mutations in the fibroblast growth factor receptor 3 (FGFR3) gene, located on chromosome 4p16.3. Although the condition follows an autosomal dominant inheritance pattern, most cases arise from de novo mutations. Constitutive activation of the FGFR3 receptor disrupts the normal regulation of chondrocyte proliferation and differentiation, leading to impaired endochondral ossification and abnormal skeletal development. These molecular alterations account for the severe phenotype observed in affected neonates [2].

## Case presentation

**Figure 1:**
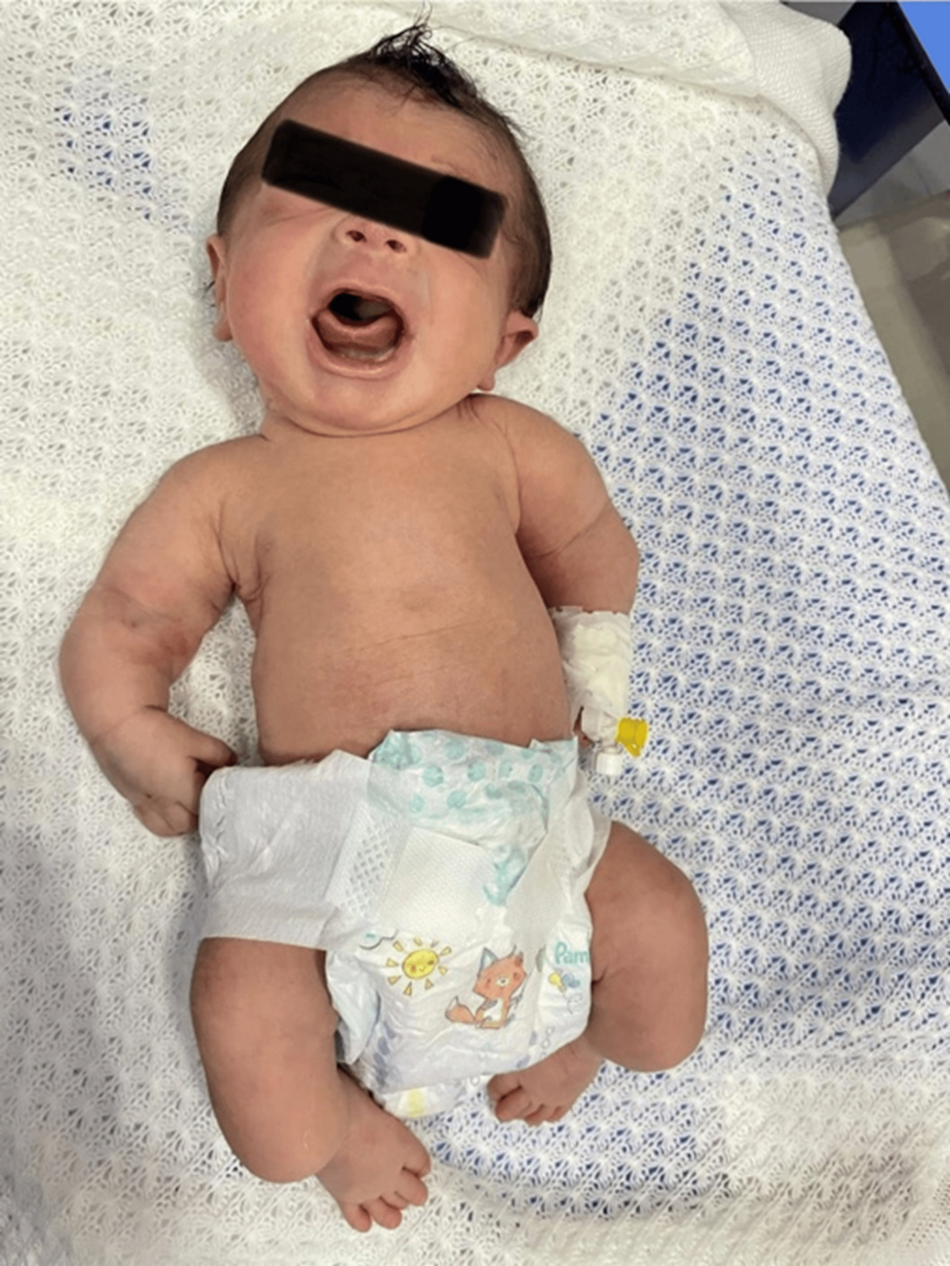
Clinical photograph demonstrating marked thoracic narrowing with disproportion between the chest and abdomen, severe limb shortening, and macrocephaly.

We report the case of a 26-year-old nulliparous woman, with no significant personal or familial medical history, in whom a second-trimester fetal anomaly scan performed at 21 weeks and 3 days of gestation revealed sonographic features suggestive of thanatophoric dysplasia type I. Ultrasound examination demonstrated marked macrocephaly, severe micromelia with bowing of the humerus and the femur, a narrow thoracic cage with a "bell-shaped" thoracoabdominal contour, short ribs, and evidence of platyspondyly. Serial ultrasound follow-up was conducted until delivery. The patient underwent a cesarean section at 39 weeks of gestation, based on the last menstrual period. The newborn male was immediately admitted to the neonatal intensive care unit (NICU) due to a polymalformative syndrome associated with respiratory distress. Initial clinical examination revealed mild central cyanosis, with an oxygen saturation of 85% on room air, which improved to 98% under 2 L/min oxygen via nasal cannula. Heart rate was stable at 120 bpm. Anthropometric measurements showed a length of 28 cm, a birth weight of 2600 g, and a head circumference of 40 cm. When interpreted using gestational-age-adjusted neonatal growth standards, the length corresponded to a Z-score well below −3, the weight approximated −2 Z-scores, and the head circumference exceeded +2 Z-scores. Crown-heel length was measured in the supine position using a neonatal length board; the extreme value reflects true skeletal shortening rather than measurement error. Dysmorphic features included macrocephaly, frontal bossing, redundant facial skin, facial edema, and a short neck. The thorax appeared narrow and disproportionate in relation to the abdomen, and all four limbs were markedly shortened (Figure 1). No clinical evidence of fractures, bone demineralization, or blue sclerae was observed, findings that helped exclude osteogenesis imperfecta and other skeletal dysplasias associated with bone fragility.

**Figure 2:**
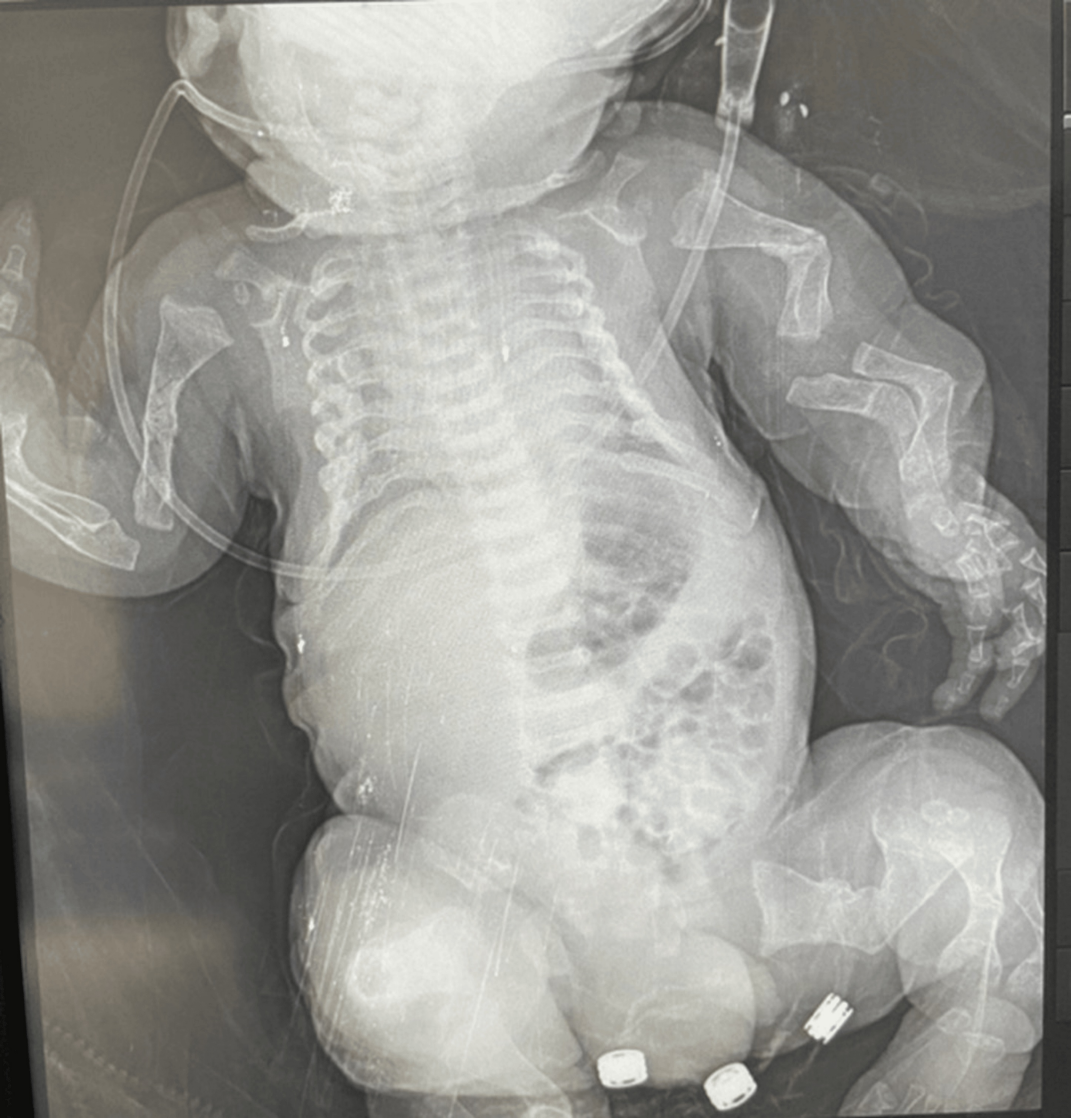
Skeletal radiograph showing markedly bowed femora with a classic “telephone-receiver” appearance, shortened ribs with a narrow thoracic cage, and generalized platyspondyly

A skeletal survey revealed markedly bowed humeri and femora with a characteristic “telephone-receiver” appearance, a narrow thoracic cage with shortened ribs, and generalized platyspondyly (Figure 2). No radiographic signs of fractures or generalized osteopenia were identified.

The radiologic and clinical findings were considered in the context of the differential diagnosis of lethal skeletal dysplasias, including osteogenesis imperfecta type II, achondrogenesis, and short rib-polydactyly syndromes. The absence of fractures, severe bone demineralization, and polydactyly, together with the classic femoral morphology, supported the diagnosis of thanatophoric dysplasia.

Based on the combination of clinical and radiological findings, the diagnosis of thanatophoric dysplasia type I was established. At the family’s request, the newborn was transferred to a private hospital in the city on day three of his life.

Follow-up information obtained from the receiving facility indicated that the neonate continued to experience progressive respiratory insufficiency consistent with severe thoracic restriction. Supportive oxygen therapy was maintained. Despite intensive supportive care, the clinical condition remained poor, reflecting the expected natural course of thanatophoric dysplasia. This outcome underscores the lethal prognosis associated with the disorder and highlights the importance of anticipatory counseling and perinatal planning.

## Discussion

Thanatophoric dysplasia is a rare congenital skeletal dysplasia, sporadic in nature, and generally fatal in the neonatal period. This condition is characterized by micromelia, a narrow conical thorax, platyspondyly (flattening of the vertebral bodies), and macrocephaly. The incidence is estimated to be between 1 in 20,000 and 1 in 50,000 live births. It results from an activating mutation in the FGFR3 gene (fibroblast growth factor receptor 3), located on the short arm of chromosome 4. The condition follows an autosomal dominant inheritance pattern. The FGFR3 receptor, involved in the regulation of cell growth, is abnormally activated by fibroblast growth factors, leading to inhibition of chondrocyte proliferation while promoting their differentiation and maturation, thus disrupting the normal process of endochondral ossification [3].

Two clinical subtypes of TD have been described. Type I, the more common form, is characterized by severe micromelia, bowed long bones (especially femora), a narrow thorax, and macrocephaly with frontal bossing. Type II, by contrast, features straight long bones and a cloverleaf skull due to premature cranial suture closure. Diagnosis is usually suspected prenatally based on characteristic ultrasound findings, including shortened limbs, narrow thoracic cavity, and macrocephaly, often as early as the second trimester [4].

The differential diagnosis of thanatophoric dysplasia includes several lethal skeletal dysplasias that may present with overlapping prenatal and radiographic features, such as homozygous achondroplasia, achondrogenesis (types 1A, 1B, and 2), SADDAN syndrome, short rib-polydactyly syndrome, osteogenesis imperfecta type II, lethal platyspondylic skeletal dysplasias, Silverman-Handmaker dyssegmental dysplasia, and campomelic dysplasia [5].

In our case, prenatal ultrasound at 21 weeks of gestation revealed classic features suggestive of TD type I, which were further supported by postnatal clinical examination and skeletal radiographs. In the absence of molecular confirmation, the diagnosis relied on the presence of highly characteristic findings. Notably, the absence of fractures or generalized bone demineralization argued against osteogenesis imperfecta, while the presence of markedly bowed long bones with classic morphology, platyspondyly, and severe thoracic narrowing strongly favored thanatophoric dysplasia. This pattern-based diagnostic reasoning is well supported in the literature and remains clinically relevant in settings where genetic testing is unavailable.

Given the high neonatal mortality associated with TD, due primarily to pulmonary hypoplasia, antenatal diagnosis allows for early parental counseling, decision-making, and perinatal planning. Neonatal management is typically supportive, with limited survival beyond the immediate postnatal period.

## Conclusions

This case highlights the critical role of prenatal ultrasound in the early detection of fetal anomalies, enabling appropriate parental counseling, optimal birth planning, and timely neonatal management when indicated. Multidisciplinary coordination between obstetricians, neonatologists, radiologists, and geneticists remains essential to provide comprehensive care and guide decision-making in the context of a lethal congenital condition.

Although molecular confirmation was not available in this case, the diagnosis was supported by highly characteristic clinical and radiologic findings. This limitation underscores the complementary role of genetic testing, when accessible, in confirming the diagnosis and refining counseling. The documented clinical course further illustrates the severe natural history of thanatophoric dysplasia and reinforces the value of early recognition for anticipatory care planning.
